# Characteristics inhibition defects of children with developmental dyscalculia: Evidence from the ERP

**DOI:** 10.3389/fpsyt.2022.877651

**Published:** 2022-10-06

**Authors:** Wang Chao, Enguo Wang, Tian Yuan, Qingqing He, Entao Zhang, Junfeng Zhao

**Affiliations:** ^1^School of Journalism and Communication, Zhengzhou University, Zhengzhou, China; ^2^Institute of Psychology and Behavior, Henan University, Kaifeng, China

**Keywords:** characteristics inhibition, negative priming effect, developmental dyscalculia, digital processing ability, P200, P300

## Abstract

Developmental dyscalculia (DD) is characterized by insufficient mathematical learning ability and weaker mathematical performance than peers who are developmentally typical. As a subtype of learning disability, developmental dyscalculia contributes to deep cognitive processing deficits, mainly manifested as a lack of numerical processing ability. This study utilized event-related potentials (ERPs) technology to examine the negative priming effects (NP) between children with and without DD. Behaviorally, trends in mean reaction time (RT) were consistent between children with and without DD under prime and control conditions. The developmental dyscalculia group and the typical developmental (TD) children group showed a significant negative priming effect. However, the magnitude of the NP was significantly different between two groups, with the magnitude being significantly higher in the TD group than the DD group. In terms of the ERPs results, there were significantly larger amplitudes of P100, P200, and P300 in the TD group than that of children with DD. At the same time, in the DD group, N100 and P300 latency were significantly delayed in some electrodes than the TD group. The results indicated that there were characteristic inhibition deficits in children with DD. Inhibition defects in children with DD might be the underlying cause of the development of digital processing ability of children with DD.

## Introduction

Developmental dyscalculia (DD), also known as mathematical disorder, is a subtype of learning disability. Previous research has illustrated that, due to the lack of mathematics learning ability, children with DD are weaker than typical developmental peers (TD) in math learning achievements (1). There are significantly lower scores on standardized math tests for children with DD than their own intelligence levels. That is not due to factors such as visual impairment, hearing impairment, and emotional disturbance. In recent years, 3–6% of school-aged children have severe difficulties in understanding number concepts, extracting computational facts, and performing computational procedures, even in normal intellectual, educational, and social contexts (2). DD affects not only a child's math performance but also their daily life and physical and mental development. Therefore, children with DD will encounter more difficulties and setbacks in many aspects, such as the quality of mathematical thinking, mathematical learning attitude, and emotion (3).

The hypothesis of digital processing deficits proposes that deficits in DD are due to difficulties in digital processing. The representation theory involves the abstract representation model (4) and the triple code model (5). The abstract representation model holds that individuals exist within an abstract digital representation system. Individuals need to transform different digital symbols into this characterization in order to conduct the psychological processing. The triple code model showed that mathematical cognitive ability was composed of three relatively independent functional components, which were the magnitude module, verbal module, and visuo-spatial module. Consequently, different modules were different in their mental representations. The different symptoms of DD illustrate the complexity of digital processing processes and the modularity of features. Some of the results supported the view of digital processing (6). However, the theoretical models are only strong hypotheses. Existing research they discovered today cannot account for all those cases in DD.

To make up for the deficiency of the theory of digital processing deficits, Ta'ir et al. (7) divided the DD into two types: the depth dyscalculia and the secondary dyscalculia. Depth dyscalculia occurs at the stage when it has a direct correlation with digital processing, which is usually not overlapped with other cognitive obstacles. Secondary dyscalculia is more in the cognitive processes of working memory, attention, inhibition, executive function, and speech cognition, which overlap with other types of cognitive impairments. To a certain extent, this classification is reasonable. Digital cognition involves multiple components and stages of cognitive processing. This means that numerical cognition includes not only general cognitive abilities (working memory, processing speed, attention, inhibition, executive function, language, etc.) but also complex components such as numerical representation, transformation, and reasoning in numerical size. It can be seen that the complexity of digital cognitive ability determines the diversity of research methods to explore the etiology of children with DD.

The working memory deficit of DD has been demonstrated by many studies. Even simple calculations require the retrieval of numerical information, so working memory, as an energy-limited mental processing system, is hard to process alongside numerical cognitive processing (8). Some studies hold that there were impairments in the speech and visuo-spatial working memory function of individuals with DD (9–11). Longitudinal studies confirmed that working memory was positively correlated with math performance (12). Wang et al. (13, 14) conducted a series of studies on the cognitive processing mechanisms of children with learning disabilities. They found that the abilities of working memory, processing speed, inhibition ability, and executive function were insufficient in children with learning disabilities. In addition, cognitive processing deficits in patients with different types of learning disabilities exist in diversity. The digital processing impairments of DD may be the most prominent symptom of deep cognitive processing deficits.

For the past few years, some people have tried to explain the deficits in the working memory of children with DD by inhibitory mechanism deficits (15). The inhibition mechanism can prevent irrelevant information from entering the working memory and quickly suppress the irrelevant information that has entered the working memory. Many studies have manifested that children with DD possess inhibition ability deficits (8, 16, 17). There is a close connection between the development of mathematical ability and the inhibition function. Some outcomes were inverse to those in the above studies. McLean and Hitch (18) took advantage of VADs Conroe Card Sorting Test to check the children with mathematics disorder of the central executive function and found that there was no significant difference between children with mathematics disorder and the TD group in the cancellation task. Therefore, there is still a controversy about the inhibition mechanism of mathematical disorder.

Distraction inhibition is an unconscious process, which is difficult to measure directly. The discovery of the negative priming effect (NP) provides an effective method to explore the mechanism of inhibition. The negative priming effect refers to the inhibitory effect when the distractor in the previous stimulus is presented as the target in the next stimulus, which is manifested as the distractor ignored in the previous display as the target stimulus in the next display. Tipper (19) first used the negative priming technique to probe the processing characteristics of the distraction. Distractions occurred in the phase of target selection. The internal representation of the disturbance was suppressed in the processing. The average response time was extended when a distraction in the priming display was targeted in a subsequent detection display. So the target stimulus acted as an ignored (inhibited) distraction. Therefore, the NP was also called the inhibitory effect of distraction. Studies (8) showed that special groups tended to show less inhibition of distraction. The NP displayed a general cognitive function. Further research is needed to characterize the neural mechanisms of distraction inhibition in children with DD.

Although some experimental results confirmed the distraction inhibition ability of children with DD, there are still many deficiencies in the previous studies. First, participants are selected. A study (15) found that developmental dyscalculia has higher comorbidity (consolidation disorder). As one of the most common comorbidities, ADHD is associated with DD, and it cannot be ruled out in some selected participants. However, the selected participants clearly influenced the results of the study in a large way. Second, the inhibition characteristics of DD are mostly from behavioral experiments. The deep processing mechanism of the NP is difficult to explain because the behavioral data can only explain the superficial phenomenon of NP. In recent years, many researchers have tried to explain the NP from the perspective of brain neural mechanism. ERP technology has temporal high resolution. Therefore, in the study of the cognitive neural mechanism of NP, ERP technology has unique advantages. Under different negative priming tasks, ERP reflects early components of response inhibition processing and reflects stimulus evaluation and whether there are differences in the amplitude of ERPs in late components related to memory. Third, the previous studies of individual distraction inhibition and interference inhibition did not distinguish between location inhibition and characteristic inhibition. Negative priming and location-dependent negative priming depend on different brain pathways. Therefore, more studies are needed to support distraction inhibition in children with DD.

The main goal of this study is to understand the neural mechanisms of characteristic inhibition of children with DD by a negative priming paradigm. Event-related potentials (ERPs) are brain potentials recorded at the scalp that reflect the synchronous firing of groups of synapses. Furthermore, ERPs, as a highly effective method in investigating the process of negative priming effect, could be used to obtain the accurate time processing. This study hypothesizes that characteristic inhibition in children with DD is defective and that there is a difference between children with DD and healthy children in the early components.

## Methods

### Participants

The study participants were selected from grade four and grade five students at three primary schools. All students took the standard Raven's Progressive Matrices (RPM) test and the Chinese Rating Scale of Basic Mathematical Competencies (BMC) at the Elementary School Level. The RPM test mainly surveyed the nonverbal intelligence of children, and the BMC test mainly inspected the difference between digital processing and mathematical calculation level. A group test was used in all tests. At the same time, the children were assessed using the Learning Disability Screening Questionnaire (LDSQ) and the Diagnostic and Statistical Manual of Mental Disorders, Fourth Edition (DSM-IV).

According to the test results, the subjects were screened by a layer-by-layer screening method. Children were assigned to the DD group if they met the following criteria: (1) The Raven intelligence level are greater than 50% (relative to the percentile rank norm); (2) The score of LDSQ was less than 65 points, using DSM-IV screening scale exclude the attention deficit hyperactivity disorder (ADHD) children; (3) Arithmetic operation ability on the Chinese Rating Scale of BMC in the Elementary School Level was less than 16.4 percentile, i.e., the arithmetic operation ability is behind the average level of the students in the same grade 1 standard deviation; (4) The recent two math test scores were ranked in the grade 30%, and the average score of Chinese performance was in the grade 50%; (5) No other mental illness and sensory disorders, no history of drug use. Children were assigned to the TD group if they met the following criteria: The Chinese and math scores were higher than those of the same grade, above 51%, no learning disabilities or ADHD, a raven intelligence level of more than 50%, no other mental illnesses and sensory disorders, and no history of drug use. Eventually, this study included 40 participants (mean age, 11 years): 20 with dyscalculia and 20 in the control group. All participants reported visual acuity or corrected visual acuity above 1.0, right-handed. Participants' information is presented in Table 1.

**Table 1:** Participants' characteristics.

	**TD group (*****n*** = **20)**		**DD group (*****n*** = **20)**			
	**M**	**SD**	**M**	**SD**	**F**	***p***
Age	10.61	0.41	11.73	0.41	0.63	0.91
Male	10	0.71	12	0.62	0.34	0.86
Female	10	0.52	8	0.81	0.45	0.77
Intelligence	85.64	3.21	75.61	4.03	1.71	0.09
Mathematical calculation	36.23	6.13	51.92	7.63	12.34	0.00
Logical reasoning	43.87	7.81	53.97	7.62	9.78	0.01

M, mean; SD, standard deviation.

Data collection has been approved by the local Ethics Committee of Henan University. All participants and their parents were provided with written informed consent before the experiment, and all participants were agreed by the guardian of the students. Financial compensation was given after participants had completed the study. The results of chi-square analysis showed that there was no significant difference in the number of participants by gender (*p* > 0.05).

### Tools

Programs written in-house with E-prime 1.1 were run on a desktop computer, which was used to present the stimuli and run the experiments. Stimuli were presented on a 14.7-inch screen (resolution: 1,440 × 900 pixels, 60 Hz). A BrainCap 32-channel Ag/AgCl electrode cap with 32-channel amplifiers (BrainProducts, Germany) was used to record the EEG data. The electrodes were arranged according to the International 10–20 expansion system, and the cap could simultaneously record the horizontal and vertical electrooculograms (HEOG and VEOG, respectively). EEG recording and analyzing systems use Ag/AgCl electrode caps to record the corresponding EEG data.

### Materials

The following 14 commonly used capital letters and Arabia figures were selected randomly: M, N, P, Q, R, T, X, 1, 2, 3, 4, 6, 7, 8. The pronunciation and shape of these selected letters and numbers are easy to distinguish to carry out the measurement of the negative priming effect.

### Experimental design

A 2 × 2 mixed experimental design was adopted. The between-subject variable is group (the DD group and the TD group). The within-subject variable is the relative location between the distraction in the priming display and the target in the detection display, which is consistent (negative priming condition) or inconsistent (control condition). The dependent variable was the average reaction time (RT) to the detection displayed.

A characteristic negative priming experimental paradigm was used. A single trial involved presenting the priming display and detection display in sequence. The priming display was a random selection of 2 symbols from 14 symbols that constitute the target and the distraction. The target and distraction term of the detection display were set according to the experimental conditions to ensure that the target and the interference term were different in each test. All the experiments included 120 trials (60 experimental trials and 60 control trials). A priming display stimulus pair in each block was presented in the vertical direction, and a detection display stimulus pair was presented in the horizontal direction. Detection display stimulus pairs appeared in the left or right visual field symmetric in order to eliminate the influence of non-symmetry in cerebral hemisphere function and inhibition of return.

### Experimental procedure

**Figure 1:**
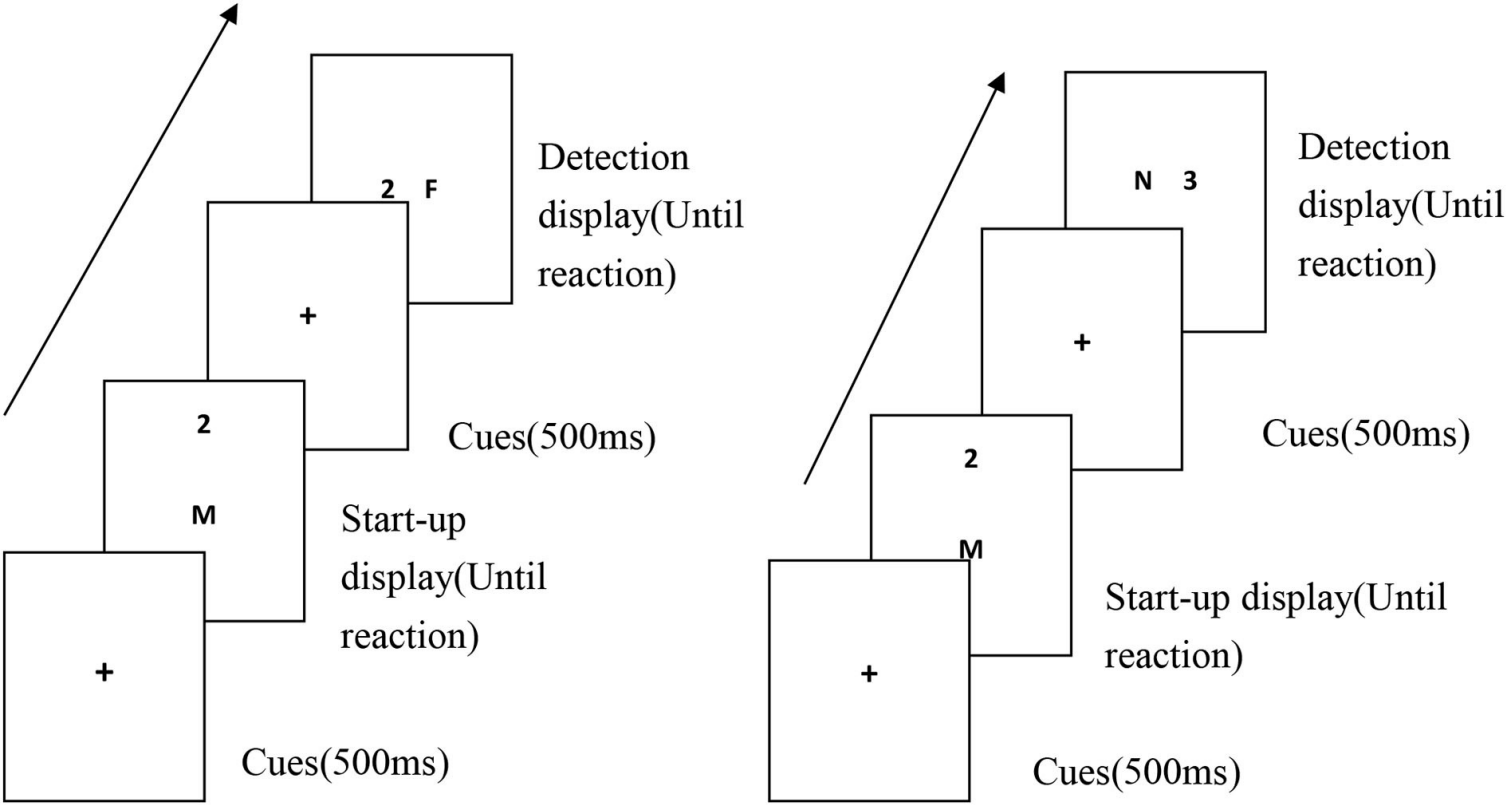
Diagram of the experimental trial procedures under the characteristic negative priming condition.

The experiments were carried out in the electroencephalography (EEG) laboratory. Each participant sat alone in a soundproofed, enclosed room for the tests. The screen was about 1 m from the participant's eyes at a horizontal viewing angle of 1.97° and a vertical viewing angle of 0.95°. The order of participation was random. The participant's two index fingers were placed on the “F” and “J” keys on the keyboard to make responses. First, the screen displays the fixation point “+,” and after the “+” disappears, letters or numbers will be randomly displayed on the screen. The subjects were asked to make a choice between numbers and English. When the English letters appeared, they were asked to press the “F” key quickly and accurately, and when the Arabic numerals appeared, they were asked to press the “J” key. Symbols in other positions do not require a keystroke. Before the experiment began, the experimenter instructed the participants to practice with the space key, using the same symbols as those in the experimental trials, which were only presented using the method for the control trials. The experiment did not formally commence until the participants completely understood the task and could make proficient responses. After 60 trials were completed, the participants rested for 1–3 min before completing the remaining trials. The specific test flow chart is shown in Figure 1.

### ERPs recording and analysis

A BrainCap 32-channel Ag/AgCl electrode cap with 32-channel amplifiers (BrainProducts, Germany) was used to record the EEG data. The electrodes were arranged according to the international 10–20 expansion system, and the cap could simultaneously record the horizontal and vertical electrooculograms (HEOG and VEOG, respectively). The ground electrode was the midpoint on the line connecting FCz and Fz. Electrodes were placed laterally to the eyes to record the HEOG and above and below the right eye to record the VEOG. The mastoid of the left ear was used as the reference electrode. The scalp resistance at each electrode was kept under 5 kΩ. The bandpass filter was 0.05–100 Hz, and the sampling frequency was 500 Hz. The average voltage at the left and right mastoids was used for offline analysis. The low-pass offline filter was 50 Hz. Amplitudes more than ±200 μV were regarded as artifacts and were excluded. The ERP observation window was −200 to 1,000 ms. The average amplitude at −200 to 0 ms was used to correct the baseline. Data accompanied by artifacts such as blinking, eye movement, and myoelectricity were excluded. EEGs from tests under different experimental conditions were categorized and superimposed to obtain the ERPs of the negative priming and control conditions.

Based on previous research (20), combined with the waveforms, topographic maps, and experimental tasks, the main ERP components of interest in this study were determined to be the P100 (90–110 ms), N100 (120–190 ms), P200 (170–220 ms), P300 (300–400 ms), and the average amplitude measurement method and the peak latency were adopted. A three-factor repeated-measure ANOVA was used to analyze the data. The factors were as follows: one between-subjects factor group (2 levels: DD vs. TD), two within-subjects factors conditions (2 levels: negative priming vs. control), and electrode location (4 levels and 12 electrodes: frontal region [F3, Fz, F4]; central region [C3, Cz, C4]; parietal region [P3, Pz, P4]; and occipital region [O1, Oz, O2]. Data were managed and ANOVA analyzed using SPSS 16.0, and Greenhouse-Geisser correction was applied.

## Results

### Behavioral results

Based on the preliminary analysis of the ERP waveforms, two participants were excluded from further analyses because of excessive blinking and other artifacts. Additionally, one participant had to be excluded because he had a response error rate that was more than 5%. One participant had to be excluded because his data exceed the average of 3 standard deviations. Thus, the valid data of 36 participants were included in the final analysis, 18 from the developmental dyscalculia group and 18 from the TD group. The accuracy rate of the two groups of subjects under different conditions was more than 90%. The average RT and the standard deviation of the detecting targets of different ability groups in two conditions are shown in Table 2.

**Table 2:** Mean reaction times (ms) of the DD and TD groups under the two conditions.

**Group**	**Priming condition**		**Control condition**	
	**M**	**SD**	**M**	**SD**
DD	788.39	142.11	767.59	126.20
TD	656.63	119.96	633.52	106.69

Data are presented as the mean ± the standard deviation.

A 2 (group) × 2 (condition) was conducted by repeated-measures analysis of variance (ANOVA) on response time data. The results showed that the main effect of group was significant (*F*_(1, 30)_ = 9.35, *p* < 0.01, η^2^_*p*_ = 0.372), whereby the average response time in the dyscalculia group was longer than that in the control group. There were significant differences in the average response times between the two groups. The main effect of condition was also significant (*F*_(1, 30)_ = 16.05, *p* < 0.001, η^2^_*p*_ = 0.451), whereby the response times under the negative priming condition was significantly prolonged compared with that in the control condition. The interaction was not significant (*F*_(1, 30)_ = 0.04, *p* > 0.05). Under different experimental conditions, variation trends of average response time of the two groups were consistent. The response times under the condition of negative priming experiment was longer than that of the response times under the control condition.

To test whether the two groups have a significant feature negative priming effect, the paired samples *t*-test was performed on the two groups of participants under the experimental condition and the control condition. The results showed that there was a significant difference in the average response time between the experimental condition and the control condition in the control group (*t* = 4.45, *df* = 16, *p* < 0.01, η^2^_*p*_ = 0.256). The control group showed significant feature negative priming effect. In the dyscalculia group, the difference was significant on the average response times under the conditions of experiment and control marginally (*t* = 2.06, *df* = 14, *p* = 0.06). In this experiment, the dyscalculia group also had a negative feature priming effect. Thus, in this experiment, the dyscalculia group and the control group had a feature negative priming effect. The results showed that the magnitude of the feature negative priming effect in the control group was significantly higher than that in the dyscalculia group. At the same time, it was found that the two groups of participants were in different experimental conditions. The average response time variation trends were consistent, which also confirmed the rationality of the design from the side.

To further investigate the feature inhibition characteristic of the different ability groups, the magnitude of the feature negative priming effect on the two groups of participants was performed by variance testing. Subtract average response times to the detecting target under the control condition from average response times to the detecting target under the negative priming condition, and the difference was the magnitude of the feature negative priming effect, which was one of the important indexes of feature inhibition. Independent samples *t*-test for the magnitude of the feature negative priming effect of the two groups was carried out. The results are shown in Table 3.

**Table 3:** The magnitude of the characteristic negative priming effect (ms) difference test (M ± SD) of the DD and TD groups.

	**DD group**		**TD group**		***F***	***p***
	**M**	**SD**	**M**	**SD**		
The magnitude of the characteristic negative priming effect	20.89	37.66	23.10	22.44	4.51	0.04

The results showed that the magnitude of the feature negative priming effect in the control group was significantly higher than that in the dyscalculia group. The results showed that the feature inhibition of the dyscalculia group was significantly lower than that of the control group. Distraction inhibition in children with dyscalculia was deficient.

### Basic features of the ERP waveforms

The ERPs recorded under the negative priming and control conditions during the feature negative priming task were categorized and superimposed, and the ERP waveforms of the negative priming task related to different ability groups were obtained. In general, the basic features of the ERPs of the two groups in the two conditions were similar, and the amplitudes under the negative priming condition were more positive than the amplitudes recorded under the control condition. First, normal visual evoked potentials were observed, beginning with the early P100 component (average latency: 106 ms) from the parietal-occipital region, followed by an early negative peak (N100; average latency: 161 ms), and then a slightly later positive peak (P200; average latency: 193 ms) at the central and frontal regions. Second, a late and positive component, the P300 (average latency: 370 ms), appeared at the parietal and central regions. From 400 ms to the end of the recording, an LPC (late positive component) that was sustained for a relatively long duration appeared at the frontal and central regions.

### Comparison of the control condition and the negative priming condition in dyscalculia group

**Figure 2:**
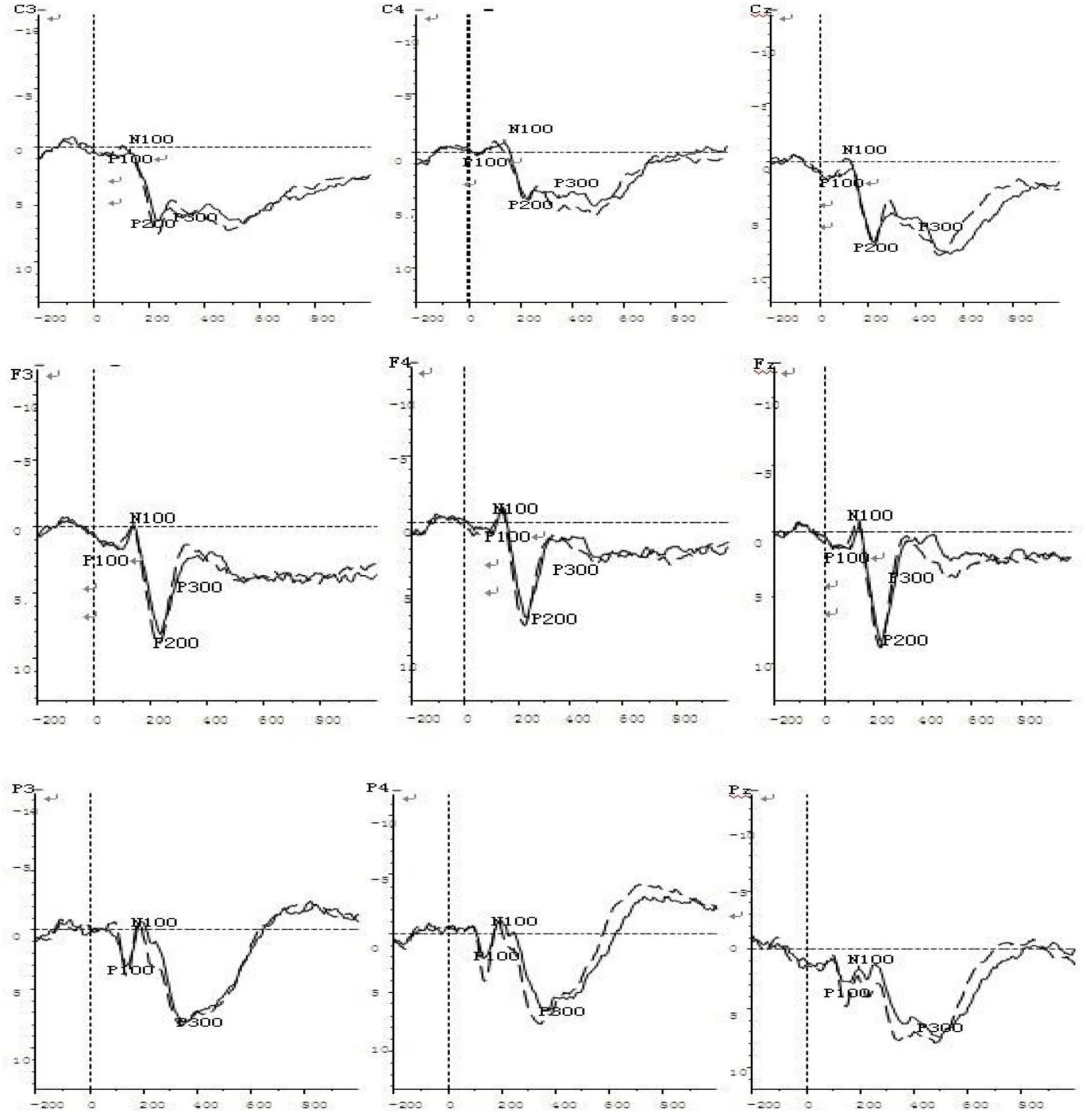
ERP waveform diagrams under the negative priming condition in the DD (solid line) and TD groups (dashed line).

Two-factor repeated measures ANOVAs were conducted for each of the main ERP components (P300, P200, P100, and N100) in dyscalculia group, with the two factors being the group (dyscalculia vs. control) and the electrode (F3, Fz, F4, C3, Cz, C4, P3, Pz, P4, O1, Oz, and O2) (refer to Figure 2).

For the P100 amplitude, the main effect of electrode was significant [*F*_(11, 506)_ = 3.32, *p* < 0.05], but the main effect of group was not significant [*F*_(1, 46)_ = 0.20, *p* = 0.66]. However, the electrode × group interaction was significant [*F*_(11, 506)_ = 5.87, *p* < 0.01, η^2^_*p*_ = 0.292]. Simple effects analyses showed significant between-subjects differences at the F3, F4, P3, P4, and PZ electrodes, with the responses of these electrodes being significantly larger of the control group than in the dyscalculia group.

For the P100 latency, the main effect of electrode was significant [*F*_(11, 528)_ = 16.53, *p* < 0.001, η^2^_*p*_ = 0.481], but the main effect of group was not significant [*F*_(1, 48)_ = 1.74, *p* = 0.19]. The electrode × group interaction was not significant [*F*_(11, 528)_ = 0.53, *p* = 0.89].

For the N100 amplitude, the main effect of electrode was significant [*F*_(11, 528)_ =16.62, *p* < 0.001, η^2^_*p*_ = 0.485], but the main effect of group was not significant [*F*_(1, 48)_ = 1.03, *p* = 0.31]. The electrode × group interaction was not significant [*F*_(11, 528)_ = 0.65, *p* = 0.78].

For the N100 latency, the main effect of electrode was significant [*F*_(11, 528)_ = 40.20, *p* < 0.001, η^2^_*p*_ = 0.531], but the main effect of group was not significant [*F*_(1, 48)_ = 1.33, *p* = 0.26]. However, the electrode × group interaction was significant [*F*_(11, 528)_ = 8.31, *p* < 0.01, η^2^_*p*_ = 0.380]. Simple effects analyses showed significant between-subjects differences at the C3, C4, and Cz electrodes, with the responses of these electrodes being significantly delayed in the dyscalculia group than the control group.

For the P200 amplitude, the main effect of electrode was significant [*F*_(11, 528)_ = 13.21, *p* < 0.01, η^2^_*p*_ = 0.331], but the main effect of group was not significant [*F*_(1, 48)_ = 1.14, *p* = 0.29]. However, the electrode × group interaction was significant [*F*_(11, 528)_ = 6.51, *p* < 0.01, η^2^_*p*_ = 0.294]. Simple effect analyses showed significant between-subjects differences at the C3, F4, F3, P3, P4, and Pz electrodes, with the responses at these electrodes being significantly larger of the control group than in the dyscalculia group.

For the P200 latency, the main effect of electrode was significant [*F*_(11, 528)_ = 87.02, *p* < 0.001, η^2^_*p*_ = 0.571], but the main effect of group was not significant [*F*_(1, 48)_ = 0.48, *p* = 0.49]. The electrode × group interaction was not significant [*F*_(11, 528)_ = 0.65, *p* = 0.79].

For the P300 amplitude, the main effect of electrode was significant [*F*_(11, 528)_ = 20.08, *p* < 0.001, η^2^_*p*_ = 0.496], but the main effect of group was not significant [*F*_(1, 48)_ = 0.66, *p* = 0.42]. However, the electrode × group interaction was significant [*F*_(11, 528)_ = 5.51, *p* < 0.01, η^2^_*p*_ = 0.182]. Simple effect analyses showed significant between-subjects differences at the C3, C4, Cz, P4, and Pz electrodes, with the responses of these electrodes being significantly larger of the control group than in the dyscalculia group.

For the P300 latency, the main effect of electrode was significant [*F*_(11, 528)_ = 3.41, *p* < 0.05, η^2^_*p*_ = 0.129], but the main effect of group was not significant [*F*_(1, 48)_ = 001, *p* = 0.93]. However, the electrode × group interaction was significant [*F*_(11, 528)_ = 2.91, *p* < 0.05, η^2^_*p*_ = 0.126]. Simple effect analyses showed significant between-subjects differences at the P4, Pz, and Cz electrodes, with the responses of these electrodes being significantly delayed in the dyscalculia group than the control group.

**Figure 3:**
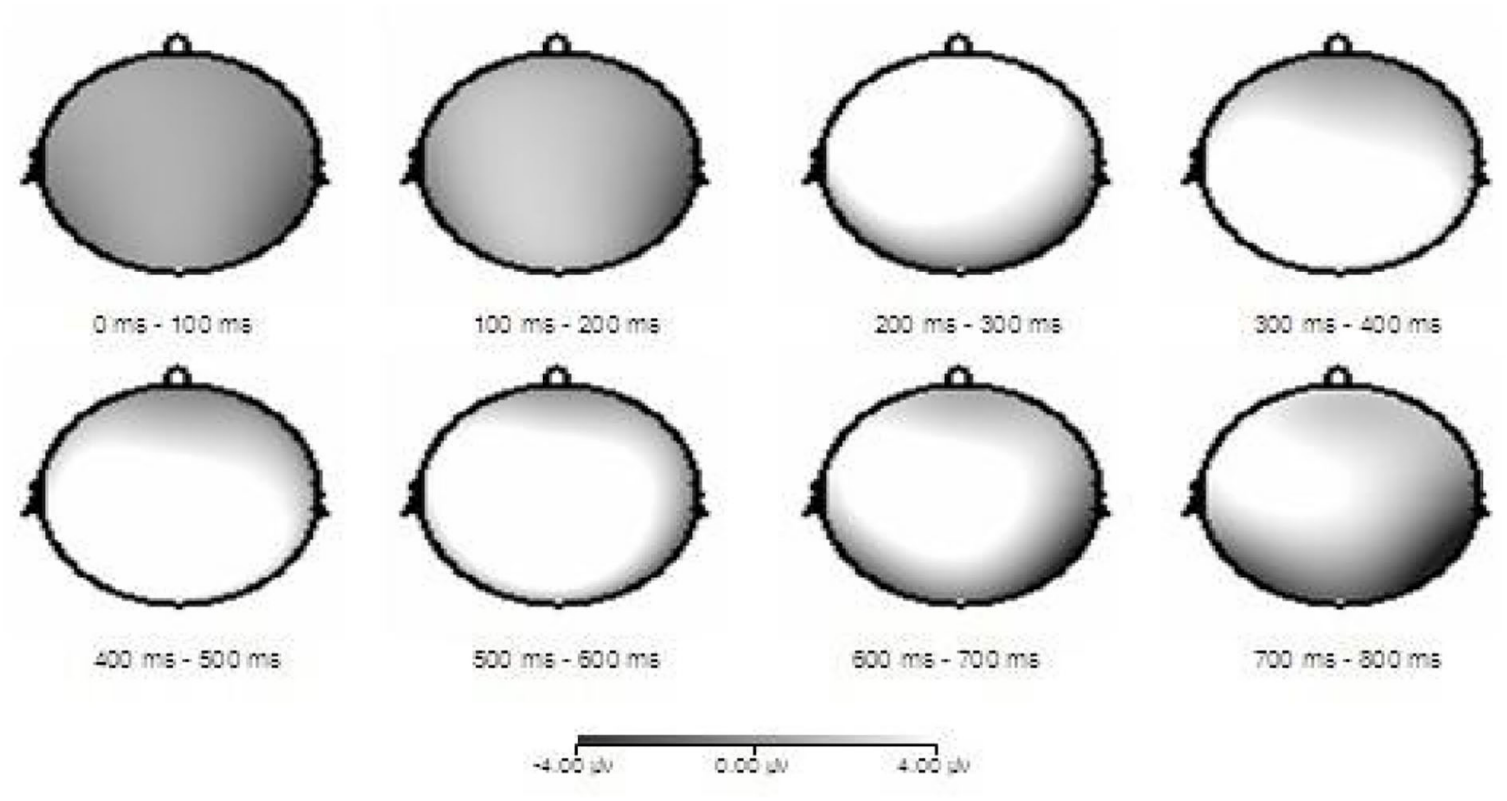
ERP differential waveform topographic maps of the DD and TD groups under the negative priming condition.

Combined with differential wave topography (Figure 3), the difference between the DD group and the TD group is mainly concentrated on the central and parietal areas. The result was consistent with the above statistical conclusions.

## Discussion

In this study, we used a feature negative priming paradigm to investigate the neural mechanisms underlying the distraction inhibition abilities of children both DD and TD. According to the behavioral data, both groups had a consistent variation trend in average RTs under the priming and control conditions. There is significant NP between the DD group and the TD group. The two groups showed longer RTs. In priming conditions, the distraction appeared as a target in the following detection display. Furthermore, it was found that the RTs were significantly longer in the DD group than in the TD group in both priming and control conditions. More importantly, there were significant differences in the magnitude of the NP between the groups, with the magnitude in the TD group being significantly higher than that in the DD group. Therefore, these results suggest that children with DD have deficits in distraction inhibition. However, there are still controversial research conclusions. Previous studies (1, 21–23) did not find significant differences between DD group and TD group in the digital processing, and the studies did not find the typical digital processing deficits in children with DD.

The ERP studies of the NP magnitude between the DD group and the TD group were reflected in main components. The P100 amplitude in the parietal and frontal areas of the TD group was significantly larger than that of the DD group. N100 latency in the central area of the DD group was significantly delayed compared to the TD group. The P200 amplitude in the parietal, central, and frontal areas of the TD group was significantly larger than that of the DD group. On part of the electrodes, the P300 amplitude in the parietal and central areas was significantly different in the two groups. In particular, the P300 amplitude of the TD group was significantly larger than that of the DD group. In part of the electrodes, the P300 latency in the parietal and central areas was significantly different between the groups. The P300 latency of the DD group was significantly delayed compared to the TD group. The P100 reflected early-stage, fast processing and selective attention to stimuli, while the N100 reflected concentrated attention to the target stimuli (15). Moreover, it suggested that the P200 component reflected the allocation of the cognitive resources that were required for evaluating the processed stimuli, while the P300 reflected the cognitive effort to process distracting stimuli.

These findings demonstrate that characteristic inhibition is deficient in children with DD. The processing time course indicated that the deficits appeared in the early processing stage. P300 is closely related to human higher psychological activities. P300 latency is delayed, which not only reflects the inhibition deficit of children with mathematics learning disabilities but also reflects the early general cognitive processing deficits. Additionally, the feature inhibition deficits of children with DD were mainly located in the parietal, central, and frontal regions of the cerebral cortex. This study was consistent with the conclusions of Bull and Scerif (8), Anobile et al. (15), Swanson (17), Jin et al. (22). However, Jin et al. (22) studied learning disabilities as a homogeneous group, so characteristic inhibition deficits in children with DD are not well defined. Our research extended the study results in this field.

The results supported the study by Cheng et al. (23). They found that the N270 latency of children with mathematics learning disabilities was higher than that of the TD group, indicating that children with learning disabilities were less able to suppress irrelevant information. The study was not consistent with the conclusions of Kathmann et al. (24) found that the characteristics of negative priming of P200 were reduced in frontal and central areas. In addition to the frontal and top regions, the parietal region was also involved in the processing of negative priming. The difference may be related to the type of materials, the number of the experimental stimuli, and the age.

In recent years, a large number of studies (8, 15, 17, 25) found that there is a close relationship between the development of mathematical ability and the inhibition function. Inhibition and attention processes are coordinated. Both determine whether the current object and processing sequence are received, and ensure that the current digital processing task is not associated with prohibited terms (26). In fact, these are intertwined in the process of processing among the inhibitory function, attention, and working memory and form the core component of the central executive function (27). Inhibition deficits affect the development of number-processing dyscalculia, which leads to lower math performance.

Inhibition deficits in children with DD can also be explained by the relationship between inhibition and working memory (13, 14, 20, 28–38). There is a reciprocal relationship between working memory and inhibition. The distraction habituation mechanism of selective attention protects working memory encoding and storage in the presence of persistent distraction. Selective attention protects working memory encoding, storage, and processing through a distraction-inhibiting mechanism under non-persistent distraction interference conditions. Bull et al. (8) study found that attention filtered the contents that entered the working memory, while the content retained in working memory played a guiding role in the attention selection process. For the typical developmental children, higher distraction inhibition can effectively prevent irrelevant information from entering into the working memory. For children with DD, inhibition ability deficits prevent more irrelevant information from entering the working memory, leading to the effects on all aspects of cognitive processing and ultimately bringing about a decline in math performance.

This study found that children with mathematics learning disabilities have specific inhibitory deficits. Training for children's inhibitory abilities can be considered to improve the academic performance in future clinical interventions. The result provides a new perspective on the intervention of children with learning disabilities.

The DD and TD groups were characterized by a negative priming effect. However, ERPs related to spatial orientation remain unclear. Future studies should utilize multi-lead source analysis to identify more regions of the brain's characteristics. Since characteristic inhibition and localization inhibition are separable, more studies are needed to explore more about the NP. Notably, dyslexic children with an average age of 11 years were selected for this study; age is an important factor affecting the amplitude of ERPs, so the findings of this study are only applicable to the current age group. In the future, the age of the research participants should be expanded to find the characteristics of the negative priming effect of dyscalculia in different age groups.

## Conclusion

There are feature inhibition deficits in children with the developmental dyscalculia, and the inhibition deficits may be one of the underlying causes of the digital processing ability of children with developmental dyscalculia.

## Data availability statement

The original contributions presented in the study are included in the article/supplementary files, further inquiries can be directed to the corresponding author/s.

## Ethics statement

The studies involving human participants were reviewed and approved by Psychology Research Ethics Committee of Henan Provincial Key Laboratory of Psychology and Behavior. Written informed consent to participate in this study was provided by the participants' legal guardian/next of kin.

## Author contributions

All authors listed have made a substantial, direct, and intellectual contribution to the work and approved it for publication.

## Funding

This study was supported by the National Social Science Foundation of China (20FJKB005), the Henan Province Philosophy and Social Science Planning Project (2020BJY010), the Henan Higher Education Teaching Reform Research and Practice Project (2021SJGLX330), and the Henan Province College Philosophy and Social Science Innovation Team Support Program (2023-CXTD-02).

## Conflict of interest

The authors declare that the research was conducted in the absence of any commercial or financial relationships that could be construed as a potential conflict of interest.

## Publisher's note

All claims expressed in this article are solely those of the authors and do not necessarily represent those of their affiliated organizations, or those of the publisher, the editors and the reviewers. Any product that may be evaluated in this article, or claim that may be made by its manufacturer, is not guaranteed or endorsed by the publisher.
